# Supplementary material for: The Evaluation of 17 Gastrointestinal Tumor Markers Reveals Prognosis Value for MUC6, CK17, and CD10 in Gallbladder-Cancer Patients
Source: Diagnostics (Basel). 2021 Jan 21;11(2):153. doi: 10.3390/diagnostics11020153 (PMC7909765; doi:10.3390/diagnostics11020153)
Supplement: Supplementary file 1 [file diagnostics-11-00153-s001.pdf]

Table S1. Antibodies used for immunohistochemical tumor-associated markers evaluation on primary gallbladder cancer biopsies.

MARKER	Supplier	CLONE	TYPE OF ANTIBODY	ISOTYPE	DILUTION	ORIGIN	POSITIVE CONTROL TISSUE
CK7	Ventana, Roche	SP 52	Monoclonal rabbit	IgG1	Prepared or pre-diluted	Mannheim, Germany	Breast
CK20	Bios SB	Ks20.8	Monoclonal mouse	IgG2a/K		Sta. Barbara, CA, USA	Colon
CK17	Cell Marque	Ks17E3	Monoclonal mouse	IgG2b		Sierra College, CA, USA	Breast
CK19	Dako Cytomation	RCK108	Monoclonal mouse	IgG1/K	1:50	Glostrup Denmark	Colon
CKLMW	Bio SB	AE1	Monoclonal mouse	IgG1		Sta. Barbara, CA, USA	Skin
CKHMW	Bio SB	34βE12	Monoclonal mouse	IgG1/K		Sta. Barbara, CA, USA	Prostate
MUC1	Bio SB	BSB-44	Monoclonal mouse	IgG1		Sta. Barbara, CA, USA	Colon
MUC2	Bio SB	BSB-45	Monoclonal mouse	IgG1		Sta. Barbara, CA, USA	Colon
MUC5AC	Bio SB	CLH2	Monoclonal mouse	IgG1		Sta. Barbara, CA, USA	Stomach
MUC6	Bio SB	CLH5	Monoclonal mouse	IgG1		Sta. Barbara, CA, USA	Stomach
CD10	Ventana, Roche	SP 67	Monoclonal rabbit	IgG1	Prepared	Mannheim, Germany	Tonsil
CEA	Bio SB	CEA 31	Monoclonal mouse	IgG1/K		Sta. Barbara, CA, USA	Colon
CA125	Bio SB	OC 125	Monoclonal mouse	IgG1/K		Sta. Barbara, CA, USA	Ovarian cancer
CDX2	Cell Marque	EPR2764 Y	Monoclonal mouse	IgG		Sierra College, CA, USA	Tonsil
VIMENTIN	Bio SB	V9	Monoclonal mouse	IgG1/K		Sta. Barbara, CA, USA	Tonsil
VILLIN	Bio SB	CWWB1	Monoclonal mouse	IgG1		Sta. Barbara, CA, USA	Colon
CLAUDIN 4	DBS		Monoclonal rabbit	-------	1:150	Pleasanton, CA, USA	Ovarian cancer

Table S2. Intensity and immunohistochemical staining pattern distribution of evaluated markers in primary gallbladder adenocarcinoma.

Marker	N° of cases	Positive cases	INTENSITY, % among + cases (# cases)			PATTERN, % among + cases (# cases)		
			Weak	Moderate	Intense	Focal	Patchy	Diffuse
CK7	168	164 (97.6%)	0.6% (1)	10.4% (17)	89% (146)	1.2% (2)	12.8% (21)	86% (141)
CK20	169	26 (15.4%)	3.8% (1)	73.1% (19)	23.1% (6)	19.2% (5)	57.7% (15)	23.1% (6)
CK17	162	118 (72.8%)	5.9% (7)	53.4% (63)	40.7% (48)	16.9% (20)	65.2% (77)	17.9% (21)
CK19	169	166 (98.2%)	0%	27% (45)	73% (121)	0.6% (1)	6.6% (11)	92.8% (154)
CKLMW	169	169 (100%)	2.4% (4)	91.7% (155)	5.9% (10)	0%	9.5% (16)	90.5% (153)
CKHMW	167	153 (91.6%)	15% (23)	49.7% (76)	35.3% (54)	15% (23)	35.9% (55)	49.1% (75)
VIMENTIN	178	7 (3.9%)	0%	28.6% (2)	71.4% (5)	0%	57.1% (4)	42.9% (3)
MUC1	170	165 (97.1%)	0%	21.8% (36)	78.2% (129)	0%	27.3% (45)	72.7% (120)
MUC2	159	5 (3.1%)	0%	20% (1)	80% (4)	20% (1)	60% (3)	20% (1)
MUC5AC	165	135 (81.8%)	0.7% (1)	20% (27)	79.3% (107)	22.2% (30)	55.6% (75)	22.2% (30)
MUC6	166	68 (41%)	0%	14.7% (10)	85.3% (58)	14.7% (10)	67.7% (46)	17.6% (12)
CDX2	165	120 (72.7%)	12.5% (15)	45% (54)	42.3% (51)	5.8% (7)	87.5% (105)	6.7% (8)
CEA	171	99 (57.9%)	0%	14.2% (14)	85.8% (85)	11.1% (11)	50.5% (50)	38.4% (38)
CA125	170	51 (30%)	13.7 % (7)	58.8% (30)	27.5% (14)	27.4% (14)	68.7% (35)	3.9% (2)
CD10	165	73 (44.2%)	4.1% (3)	45.2% (33)	50.7% (37)	11% (8)	82.2% (60)	6.8% (5)
CLAUDIN4	164	132 (81%)	3.8% (5)	73.5% (97)	22.7% (30)	1.5% (2)	63.6% (84)	34.9% (46)
VILLIN	173	81 (46.8%)	13.6% (11)	56.8% (46)	29.6% (24)	2.5% (2)	77.8% (63)	19.7% (16)

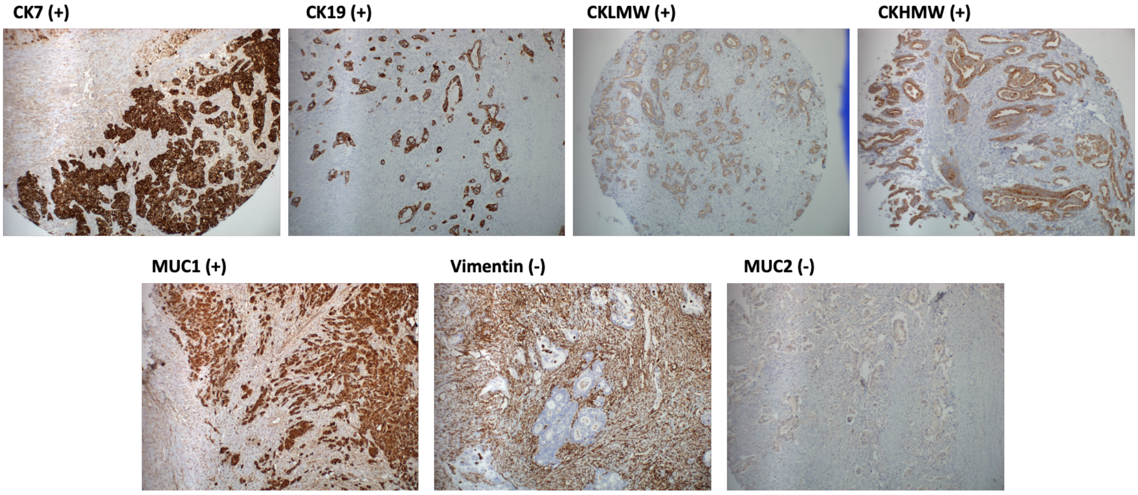

Figure S1: Examples of immunohistochemistry staining for tumor-associated markers expressed with high or low frequency in GBC patients. Representative photographs at 10X magnification are shown.

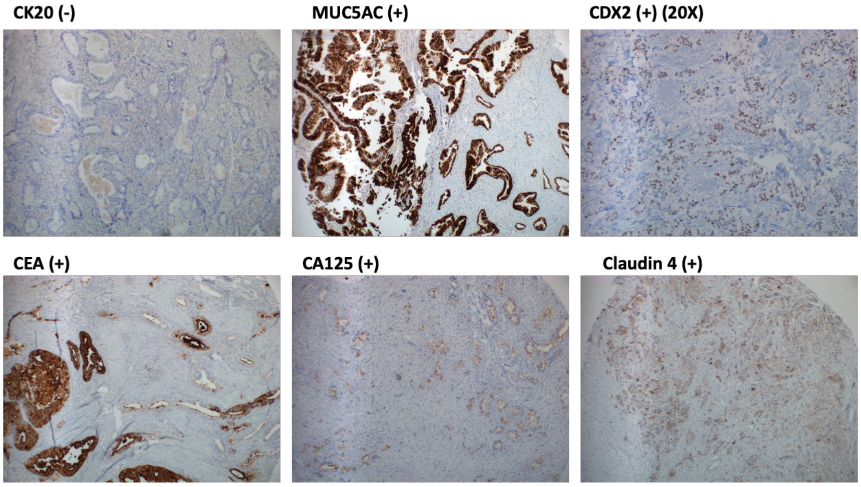

Figure S2: Examples of immunohistochemistry staining for tumor-associated markers do not associated with prognosis in our GBC patient cohort. Representative photographs at 10X magnification are shown for all markers but not for CDX2 (20X magnification).

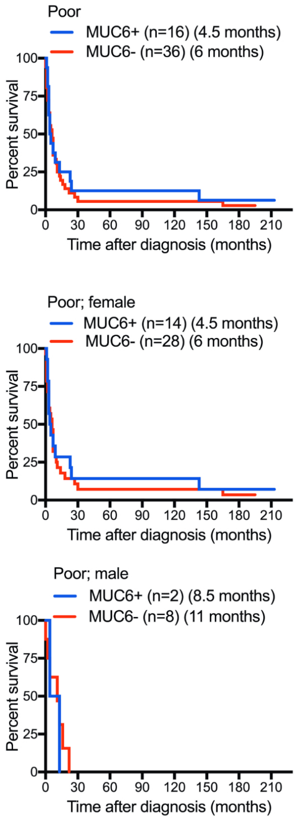

Figure S3: MUC6 tumor expression did not correlate with overall survival of patients with poorly differentiated tumors. Kaplan-Meier post-diagnosis overall survival (OS) estimation of GBC patients with poorly differentiated tumors according to MUC6 tumor expression pattern (upper, total patients; middle, female patients; bottom, male patients). Each graph showed the number of patients included in each group (n) and the median OS time in months.

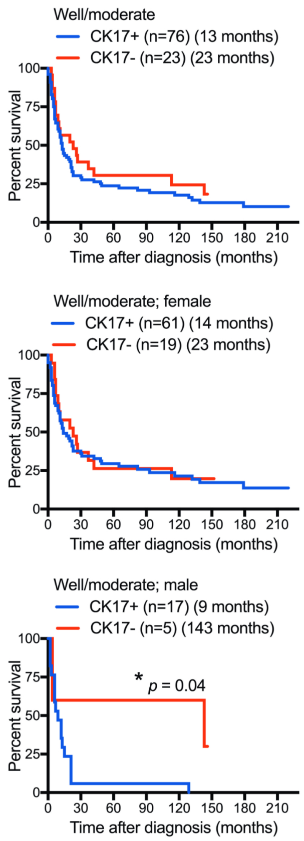

Figure S4: CK17 tumor expression correlates with a worse prognosis in male GBC patients with well to moderately differentiated tumors. Kaplan-Meier post-diagnosis overall survival (OS) estimation of GBC patients with well to moderately differentiated tumors according to CK17 tumor expression pattern (upper, total patients; middle, female patients; bottom, male patients). Each graph showed the number of patients included in each group (n) and the median OS time in months. Only p values < 0.05 are shown.
